# Aqua­(2,6-dihy­droxy­benzoato-κ*O* ^1^)bis­(1,10-phenanthroline-κ^2^ *N*,*N*′)manganese(II) 2,6-dihy­droxy­benzoate hemihydrate

**DOI:** 10.1107/S1600536812022155

**Published:** 2012-05-26

**Authors:** Quanwei Li, Li He, Hongxiao Jin

**Affiliations:** a College of Materials Science and Engineering, China Jiliang University, Hangzhou 310018, People’s Republic of China

## Abstract

In the complex cation of the title compound, [Mn(C_7_H_5_O_4_)(C_12_H_8_N_2_)_2_(H_2_O)](C_7_H_5_O_4_)·0.5H_2_O, the Mn^II^ atom has a six-coordinate octa­hedral environment defined by one carboxyl­ate O atom belonging to a 2,6-dihy­droxy­benzoate (DHB) ligand, four N atoms from two chelating 1,10-phenanthroline mol­ecules and one water mol­ecule. The lattice water mol­ecule lies on a twofold rotation axis. Intra­molecular O—H⋯O hydrogen bonds are present in the DHB anions and complex cations. Inter­molecular O—H⋯O hydrogen bonds link two cations, two anions and one water mol­ecule into a dimer. π–π inter­actions between the pyridine and benzene rings and between the benzene rings are also observed [centroid–centroid distances = 3.7774 (16), 3.7912 (16) and 3.7310 (17) Å].

## Related literature

For related structures of dihy­droxy­benzoate manganese(II) complexes, see: Garribba *et al.* (2004 ▶). For the structure of a neodymium(III) complex containing 2,6-dihy­droxy­benzoate ligands, see: Zheng *et al.* (2010 ▶).
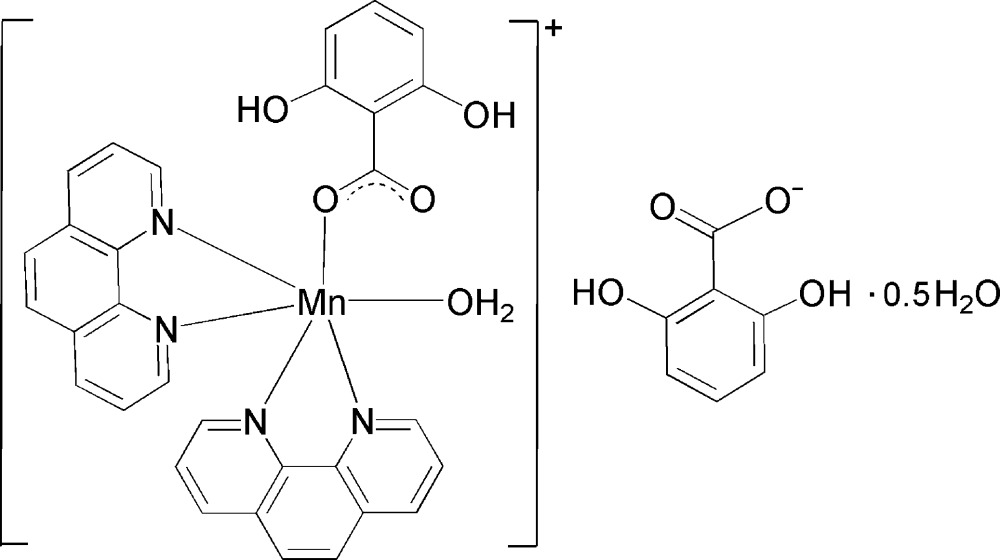


## Experimental

### 

#### Crystal data

[Mn(C_7_H_5_O_4_)(C_12_H_8_N_2_)_2_(H_2_O)](C_7_H_5_O_4_)·0.5H_2_O
*M* *_r_* = 748.59
Orthorhombic, 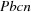

*a* = 30.0648 (7) Å
*b* = 8.2468 (2) Å
*c* = 27.3311 (6) Å
*V* = 6776.4 (3) Å^3^
*Z* = 8
Cu *K*α radiation
μ = 3.73 mm^−1^
*T* = 298 K
0.36 × 0.32 × 0.30 mm

#### Data collection

Oxford Diffraction Gemini S Ultra diffractometer
Absorption correction: multi-scan (*CrysAlis RED*; Oxford Diffraction, 2006 ▶) *T*_min_ = 0.347, *T*_max_ = 0.401
28472 measured reflections
6613 independent reflections
5152 reflections with *I* > 2σ(*I*)
*R*_int_ = 0.035

#### Refinement

*R*[*F* ^2^ > 2σ(*F* ^2^)] = 0.039
*wR*(*F* ^2^) = 0.107
*S* = 1.08
6613 reflections
478 parameters
H-atom parameters constrained
Δρ_max_ = 0.29 e Å^−3^
Δρ_min_ = −0.21 e Å^−3^

### 

Data collection: *CrysAlis CCD* (Oxford Diffraction, 2006 ▶); cell refinement: *CrysAlis RED* (Oxford Diffraction, 2006 ▶); data reduction: *CrysAlis RED*; program(s) used to solve structure: *SHELXS97* (Sheldrick, 2008 ▶); program(s) used to refine structure: *SHELXL97* (Sheldrick, 2008 ▶); molecular graphics: *ORTEP-3* (Farrugia, 1997 ▶); software used to prepare material for publication: *SHELXL97*.

## Supplementary Material

Crystal structure: contains datablock(s) I, global. DOI: 10.1107/S1600536812022155/hy2545sup1.cif

Structure factors: contains datablock(s) I. DOI: 10.1107/S1600536812022155/hy2545Isup2.hkl

Additional supplementary materials:  crystallographic information; 3D view; checkCIF report

## Figures and Tables

**Table 1:** Hydrogen-bond geometry (Å, °)

*D*—H⋯*A*	*D*—H	H⋯*A*	*D*⋯*A*	*D*—H⋯*A*
O3—H3⋯O1	0.82	1.83	2.552 (2)	147
O4—H4⋯O2	0.82	1.79	2.525 (3)	148
O7—H7⋯O6	0.82	1.79	2.518 (4)	147
O8—H8⋯O5	0.82	1.74	2.474 (3)	149
O9—H9*A*⋯O2	0.85	1.90	2.735 (2)	169
O9—H9*B*⋯O6	0.85	1.80	2.648 (3)	171
O10—H10*A*⋯O5	0.85	2.07	2.890 (3)	162
O10—H10*B*⋯O5^i^	0.85	2.09	2.890 (3)	156

Symmetry code: (i) 
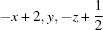
.

**Fig. 1.**
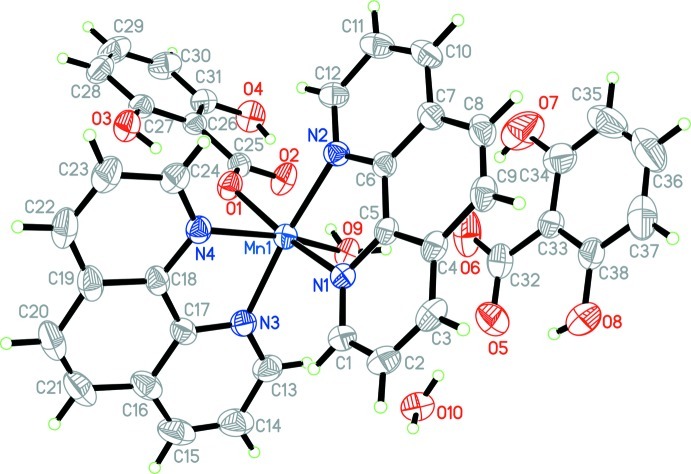
The molecular structure of the title compound. Displacement ellipsoids are drawn at the 30% probability level.

## supplementary crystallographic information

### Comment

Dihydroxybenzoate is a good ligand in the construction of coordination complexes. Several dihydroxybenzoate manganese(II) complexes have been reported (Garribba *et al.*, 2004). In our previous paper, we reported a mononuclear neodymium(III) complex containing 2,6-dihydroxybenzoate ligands (Zheng *et al.*, 2010). Herein, we reported the synthesis and crystal structure of a mononuclear manganese(II) complex formed by 2,6-dihydroxybenzoate and 1,10-phenanthroline ligands (Fig. 1).

### Experimental

All reagents were commercially available and of analytical grade. Mn(CH_3_CO_2_)_2_.6H_2_O (0.123 g, 0.5 mmol), 2,6-dihydroxybenzoic acid (0.074 g, 0.5 mmol), 1,10-phenanthroline (0.090 g, 0.5 mmol) and NaHCO_3_ (0.042 g, 0.5 mmol) were dissolved in a water–ethanol solution (10 ml, 1:1 v/v). The solution was refluxed for 4 h and filtered after cooling to room temperature. Orange single crystals were obtained from the filtrate after 14 days.

### Refinement

H atoms were positioned geometrically and refined as riding, with C—H = 0.93 and O—H = 0.82 Å and *U*_iso_(H) = 1.2*U*_eq_(C) or 1.5*U*_eq_(O). Water H atoms were located from a difference Fourier map and refined as riding, with O—H = 0.85 Å and *U*_iso_(H) = 1.5*U*_eq_(O).

### Crystal data

**Table:**

[Mn(C_7_H_5_O_4_)(C_12_H_8_N_2_)_2_(H_2_O)](C_7_H_5_O_4_)·0.5H_2_O	*F*(000) = 3088
*M**_r_* = 748.59	*D*_x_ = 1.468 Mg m^−^^3^
Orthorhombic, *P**b**c**n*	Cu *K*α radiation, λ = 1.54184 Å
Hall symbol: -P 2n 2ab	Cell parameters from 7956 reflections
*a* = 30.0648 (7) Å	θ = 2.9–72.2°
*b* = 8.2468 (2) Å	µ = 3.73 mm^−^^1^
*c* = 27.3311 (6) Å	*T* = 298 K
*V* = 6776.4 (3) Å^3^	Block, orange
*Z* = 8	0.36 × 0.32 × 0.30 mm

### Data collection

**Table:**

Oxford Diffraction Gemini S Ultra diffractometer	6613 independent reflections
Radiation source: Enhance Ultra (Cu) X-ray Source	5152 reflections with *I* > 2σ(*I*)
Mirror monochromator	*R*_int_ = 0.035
Detector resolution: 15.9149 pixels mm^-1^	θ_max_ = 72.4°, θ_min_ = 2.9°
ω scans	*h* = −36→37
Absorption correction: multi-scan (*CrysAlis RED*; Oxford Diffraction, 2006)	*k* = −6→10
*T*_min_ = 0.347, *T*_max_ = 0.401	*l* = −33→33
28472 measured reflections	

### Refinement

**Table:**

Refinement on *F*^2^	Primary atom site location: structure-invariant direct methods
Least-squares matrix: full	Secondary atom site location: difference Fourier map
*R*[*F*^2^ > 2σ(*F*^2^)] = 0.039	Hydrogen site location: inferred from neighbouring sites
*wR*(*F*^2^) = 0.107	H-atom parameters constrained
*S* = 1.08	*w* = 1/[σ^2^(*F*_o_^2^) + (0.0368*P*)^2^ + 2.5648*P*] where *P* = (*F*_o_^2^ + 2*F*_c_^2^)/3
6613 reflections	(Δ/σ)_max_ = 0.001
478 parameters	Δρ_max_ = 0.29 e Å^−^^3^
0 restraints	Δρ_min_ = −0.21 e Å^−^^3^

### Special details

**Table:**

Geometry. All e.s.d.'s (except the e.s.d. in the dihedral angle between two l.s. planes) are estimated using the full covariance matrix. The cell e.s.d.'s are taken into account individually in the estimation of e.s.d.'s in distances, angles and torsion angles; correlations between e.s.d.'s in cell parameters are only used when they are defined by crystal symmetry. An approximate (isotropic) treatment of cell e.s.d.'s is used for estimating e.s.d.'s involving l.s. planes.
Refinement. Refinement of *F*^2^ against ALL reflections. The weighted *R*-factor *wR* and goodness of fit *S* are based on *F*^2^, conventional *R*-factors *R* are based on *F*, with *F* set to zero for negative *F*^2^. The threshold expression of *F*^2^ > σ(*F*^2^) is used only for calculating *R*-factors(gt) *etc*. and is not relevant to the choice of reflections for refinement. *R*-factors based on *F*^2^ are statistically about twice as large as those based on *F*, and *R*- factors based on ALL data will be even larger.

### Fractional atomic coordinates and isotropic or equivalent isotropic displacement parameters (Å^2^)

**Table:**

	*x*	*y*	*z*	*U*_iso_*/*U*_eq_	Occ. (<1)
Mn1	0.904768 (12)	0.27378 (4)	0.113182 (13)	0.04739 (11)	
O1	0.87384 (5)	0.43792 (19)	0.06526 (6)	0.0560 (4)	
O2	0.85364 (7)	0.6491 (2)	0.11035 (6)	0.0708 (5)	
O3	0.84933 (7)	0.4138 (2)	−0.02385 (6)	0.0686 (5)	
H3	0.8612	0.3857	0.0018	0.103*	
O4	0.80413 (7)	0.8670 (2)	0.07505 (8)	0.0777 (5)	
H4	0.8199	0.8209	0.0952	0.116*	
O5	0.92987 (8)	0.2412 (3)	0.28186 (9)	0.0983 (7)	
O6	0.87072 (10)	0.3792 (3)	0.25639 (8)	0.1094 (9)	
O7	0.79336 (9)	0.2862 (4)	0.27780 (12)	0.1330 (12)	
H7	0.8128	0.3477	0.2678	0.200*	
O8	0.92100 (7)	−0.0039 (4)	0.33313 (10)	0.0994 (7)	
H8	0.9335	0.0716	0.3193	0.149*	
O9	0.90225 (5)	0.4442 (2)	0.16807 (6)	0.0584 (4)	
H9A	0.8864	0.5147	0.1536	0.088*	
H9B	0.8944	0.4169	0.1968	0.088*	
O10	1.0000	0.4563 (3)	0.2500	0.0771 (8)	
H10A	0.9757	0.4114	0.2584	0.085*	0.50
H10B	1.0160	0.3729	0.2452	0.095*	0.50
N1	0.92663 (6)	0.0806 (2)	0.15615 (6)	0.0474 (4)	
N2	0.84369 (6)	0.1748 (2)	0.13519 (7)	0.0474 (4)	
N3	0.96907 (6)	0.3514 (2)	0.09744 (7)	0.0545 (5)	
N4	0.91591 (6)	0.1556 (2)	0.04627 (7)	0.0497 (4)	
C1	0.96754 (8)	0.0270 (3)	0.16341 (9)	0.0569 (6)	
H1	0.9910	0.0849	0.1498	0.068*	
C2	0.97727 (9)	−0.1116 (3)	0.19038 (10)	0.0659 (7)	
H2	1.0065	−0.1476	0.1932	0.079*	
C3	0.94342 (9)	−0.1943 (3)	0.21265 (9)	0.0628 (7)	
H3A	0.9495	−0.2860	0.2313	0.075*	
C4	0.89980 (8)	−0.1404 (3)	0.20734 (8)	0.0514 (5)	
C5	0.89295 (7)	−0.0040 (3)	0.17738 (8)	0.0453 (5)	
C6	0.84838 (7)	0.0510 (3)	0.16779 (8)	0.0446 (5)	
C7	0.81257 (8)	−0.0247 (3)	0.19095 (9)	0.0535 (6)	
C8	0.82074 (9)	−0.1589 (3)	0.22284 (9)	0.0606 (6)	
H5	0.7970	−0.2076	0.2389	0.073*	
C9	0.86214 (10)	−0.2160 (3)	0.23004 (9)	0.0607 (6)	
H9	0.8664	−0.3059	0.2500	0.073*	
C10	0.76990 (8)	0.0347 (3)	0.18059 (10)	0.0653 (7)	
H10	0.7451	−0.0087	0.1962	0.078*	
C11	0.76506 (8)	0.1563 (3)	0.14745 (11)	0.0667 (7)	
H11	0.7369	0.1949	0.1397	0.080*	
C12	0.80263 (8)	0.2231 (3)	0.12502 (10)	0.0575 (6)	
H12	0.7987	0.3048	0.1020	0.069*	
C13	0.99607 (9)	0.4387 (3)	0.12563 (11)	0.0688 (7)	
H13	0.9860	0.4730	0.1561	0.083*	
C14	1.03887 (10)	0.4802 (4)	0.11101 (14)	0.0869 (10)	
H14	1.0573	0.5380	0.1320	0.104*	
C15	1.05357 (10)	0.4364 (4)	0.06625 (14)	0.0870 (10)	
H15	1.0820	0.4659	0.0561	0.104*	
C16	1.02588 (9)	0.3460 (3)	0.03496 (11)	0.0691 (8)	
C17	0.98391 (8)	0.3022 (3)	0.05320 (9)	0.0539 (6)	
C18	0.95485 (8)	0.2018 (3)	0.02497 (8)	0.0522 (5)	
C19	0.96681 (10)	0.1560 (3)	−0.02248 (10)	0.0664 (7)	
C20	1.00934 (12)	0.2082 (4)	−0.04083 (12)	0.0833 (10)	
H20	1.0176	0.1800	−0.0725	0.100*	
C21	1.03732 (11)	0.2962 (4)	−0.01362 (13)	0.0864 (10)	
H21	1.0648	0.3256	−0.0265	0.104*	
C22	0.93619 (12)	0.0618 (4)	−0.04863 (10)	0.0792 (9)	
H22	0.9426	0.0302	−0.0805	0.095*	
C23	0.89707 (11)	0.0165 (4)	−0.02745 (10)	0.0754 (8)	
H23	0.8766	−0.0458	−0.0447	0.091*	
C24	0.88821 (9)	0.0650 (3)	0.02050 (9)	0.0613 (6)	
H24	0.8617	0.0322	0.0349	0.074*	
C25	0.85339 (7)	0.5720 (3)	0.07086 (8)	0.0492 (5)	
C26	0.82837 (7)	0.6362 (3)	0.02839 (8)	0.0483 (5)	
C27	0.82782 (8)	0.5569 (3)	−0.01695 (9)	0.0552 (6)	
C28	0.80537 (10)	0.6231 (4)	−0.05679 (10)	0.0742 (8)	
H28	0.8059	0.5715	−0.0870	0.089*	
C29	0.78247 (11)	0.7658 (4)	−0.05062 (13)	0.0867 (10)	
H29	0.7672	0.8094	−0.0771	0.104*	
C30	0.78140 (10)	0.8462 (4)	−0.00658 (13)	0.0815 (9)	
H30	0.7653	0.9420	−0.0032	0.098*	
C31	0.80450 (8)	0.7826 (3)	0.03246 (10)	0.0598 (6)	
C32	0.88844 (12)	0.2651 (4)	0.27998 (10)	0.0725 (8)	
C33	0.85863 (8)	0.1496 (3)	0.30562 (9)	0.0589 (6)	
C34	0.81229 (11)	0.1636 (5)	0.30321 (12)	0.0851 (10)	
C35	0.78520 (13)	0.0503 (7)	0.32548 (16)	0.1192 (17)	
H35	0.7544	0.0592	0.3233	0.143*	
C36	0.80394 (18)	−0.0743 (6)	0.35054 (16)	0.1225 (18)	
H36	0.7855	−0.1489	0.3660	0.147*	
C37	0.84936 (15)	−0.0940 (5)	0.35391 (12)	0.0987 (12)	
H37	0.8615	−0.1805	0.3712	0.118*	
C38	0.87655 (10)	0.0180 (4)	0.33094 (9)	0.0684 (7)	

### Atomic displacement parameters (Å^2^)

**Table:**

	*U*^11^	*U*^22^	*U*^33^	*U*^12^	*U*^13^	*U*^23^
Mn1	0.04736 (19)	0.0504 (2)	0.04444 (19)	0.00031 (15)	0.00056 (15)	0.00292 (16)
O1	0.0669 (10)	0.0543 (9)	0.0468 (9)	0.0109 (8)	−0.0036 (7)	0.0033 (7)
O2	0.0984 (14)	0.0633 (11)	0.0505 (10)	0.0151 (10)	−0.0119 (9)	−0.0060 (9)
O3	0.0799 (13)	0.0720 (12)	0.0540 (10)	0.0045 (10)	−0.0015 (9)	−0.0062 (9)
O4	0.0936 (15)	0.0586 (11)	0.0808 (13)	0.0140 (10)	0.0004 (11)	−0.0023 (10)
O5	0.0815 (15)	0.1182 (19)	0.0952 (17)	−0.0267 (14)	0.0113 (13)	0.0121 (14)
O6	0.171 (3)	0.0844 (15)	0.0724 (14)	0.0355 (17)	0.0304 (15)	0.0189 (13)
O7	0.108 (2)	0.174 (3)	0.118 (2)	0.075 (2)	−0.0212 (18)	−0.027 (2)
O8	0.0818 (14)	0.128 (2)	0.0880 (17)	0.0105 (14)	−0.0200 (13)	0.0289 (15)
O9	0.0723 (11)	0.0570 (10)	0.0457 (9)	0.0026 (8)	−0.0053 (8)	0.0004 (7)
O10	0.0698 (17)	0.0724 (17)	0.089 (2)	0.000	−0.0111 (15)	0.000
N1	0.0442 (9)	0.0544 (11)	0.0437 (10)	0.0021 (8)	−0.0010 (8)	0.0026 (8)
N2	0.0440 (9)	0.0491 (10)	0.0490 (10)	0.0025 (8)	0.0003 (8)	−0.0027 (8)
N3	0.0530 (11)	0.0576 (12)	0.0531 (11)	−0.0050 (9)	−0.0015 (9)	0.0098 (9)
N4	0.0531 (11)	0.0502 (10)	0.0458 (10)	0.0051 (8)	0.0005 (8)	0.0025 (9)
C1	0.0472 (12)	0.0689 (16)	0.0545 (14)	0.0040 (11)	−0.0039 (10)	0.0059 (12)
C2	0.0588 (15)	0.0690 (16)	0.0697 (16)	0.0114 (13)	−0.0140 (13)	0.0055 (14)
C3	0.0777 (18)	0.0551 (14)	0.0556 (14)	0.0059 (12)	−0.0190 (13)	0.0057 (12)
C4	0.0671 (14)	0.0453 (12)	0.0418 (11)	−0.0031 (11)	−0.0065 (10)	−0.0021 (10)
C5	0.0536 (12)	0.0446 (11)	0.0376 (10)	−0.0004 (9)	−0.0017 (9)	−0.0038 (9)
C6	0.0475 (11)	0.0453 (11)	0.0410 (11)	−0.0021 (9)	0.0018 (9)	−0.0044 (9)
C7	0.0549 (13)	0.0543 (13)	0.0515 (13)	−0.0094 (10)	0.0072 (10)	−0.0091 (11)
C8	0.0713 (17)	0.0588 (15)	0.0517 (14)	−0.0183 (13)	0.0083 (12)	−0.0017 (12)
C9	0.0869 (19)	0.0495 (13)	0.0458 (13)	−0.0134 (13)	−0.0009 (12)	0.0016 (11)
C10	0.0493 (13)	0.0684 (17)	0.0782 (18)	−0.0085 (12)	0.0166 (12)	−0.0151 (14)
C11	0.0444 (13)	0.0670 (17)	0.089 (2)	0.0065 (11)	0.0021 (13)	−0.0127 (15)
C12	0.0478 (13)	0.0558 (14)	0.0689 (16)	0.0070 (11)	−0.0025 (11)	−0.0021 (12)
C13	0.0621 (16)	0.0735 (17)	0.0708 (17)	−0.0157 (14)	−0.0115 (13)	0.0085 (14)
C14	0.0634 (18)	0.090 (2)	0.107 (3)	−0.0190 (16)	−0.0103 (18)	0.015 (2)
C15	0.0509 (16)	0.087 (2)	0.124 (3)	−0.0093 (15)	0.0057 (18)	0.031 (2)
C16	0.0559 (15)	0.0659 (16)	0.086 (2)	0.0089 (13)	0.0175 (14)	0.0266 (15)
C17	0.0500 (12)	0.0536 (13)	0.0581 (14)	0.0044 (10)	0.0064 (11)	0.0157 (11)
C18	0.0584 (13)	0.0512 (13)	0.0469 (12)	0.0127 (11)	0.0093 (10)	0.0108 (10)
C19	0.0823 (18)	0.0654 (16)	0.0516 (14)	0.0235 (14)	0.0135 (13)	0.0115 (13)
C20	0.096 (2)	0.086 (2)	0.0675 (19)	0.0298 (19)	0.0383 (18)	0.0227 (17)
C21	0.074 (2)	0.088 (2)	0.097 (2)	0.0197 (17)	0.0397 (19)	0.0337 (19)
C22	0.109 (2)	0.083 (2)	0.0460 (14)	0.0301 (18)	0.0036 (15)	−0.0038 (14)
C23	0.094 (2)	0.0752 (19)	0.0576 (16)	0.0119 (16)	−0.0109 (15)	−0.0135 (14)
C24	0.0660 (15)	0.0605 (15)	0.0574 (14)	0.0032 (12)	−0.0063 (12)	−0.0045 (12)
C25	0.0510 (12)	0.0479 (12)	0.0487 (12)	−0.0033 (10)	0.0011 (10)	0.0057 (10)
C26	0.0443 (11)	0.0501 (12)	0.0506 (12)	−0.0069 (9)	−0.0020 (9)	0.0073 (10)
C27	0.0499 (13)	0.0599 (14)	0.0559 (14)	−0.0088 (11)	−0.0032 (11)	0.0081 (11)
C28	0.0814 (19)	0.085 (2)	0.0559 (15)	−0.0150 (16)	−0.0181 (14)	0.0093 (15)
C29	0.088 (2)	0.086 (2)	0.086 (2)	−0.0039 (18)	−0.0383 (18)	0.0229 (18)
C30	0.0746 (19)	0.0694 (18)	0.100 (2)	0.0107 (15)	−0.0217 (17)	0.0168 (18)
C31	0.0582 (14)	0.0546 (14)	0.0666 (16)	−0.0017 (11)	−0.0034 (12)	0.0059 (12)
C32	0.094 (2)	0.0728 (18)	0.0509 (15)	0.0036 (16)	0.0064 (14)	−0.0024 (14)
C33	0.0607 (14)	0.0715 (16)	0.0445 (12)	−0.0013 (12)	−0.0018 (11)	−0.0096 (12)
C34	0.0704 (19)	0.119 (3)	0.0663 (18)	0.0132 (19)	−0.0021 (15)	−0.0299 (19)
C35	0.074 (2)	0.185 (5)	0.099 (3)	−0.037 (3)	0.029 (2)	−0.059 (3)
C36	0.135 (4)	0.144 (4)	0.088 (3)	−0.076 (3)	0.042 (3)	−0.031 (3)
C37	0.143 (4)	0.091 (2)	0.0624 (19)	−0.029 (2)	0.007 (2)	0.0038 (17)
C38	0.0748 (18)	0.0837 (19)	0.0467 (14)	−0.0116 (15)	−0.0032 (12)	0.0029 (13)

### Geometric parameters (Å, º)

**Table:**

Mn1—O9	2.0567 (16)	C10—C11	1.359 (4)
Mn1—N3	2.0812 (19)	C10—H10	0.9300
Mn1—N1	2.0851 (18)	C11—C12	1.398 (4)
Mn1—N2	2.0977 (18)	C11—H11	0.9300
Mn1—N4	2.0993 (19)	C12—H12	0.9300
Mn1—O1	2.1005 (15)	C13—C14	1.390 (4)
O1—C25	1.275 (3)	C13—H13	0.9300
O2—C25	1.253 (3)	C14—C15	1.350 (5)
O3—C27	1.359 (3)	C14—H14	0.9300
O3—H3	0.8200	C15—C16	1.407 (4)
O4—C31	1.356 (3)	C15—H15	0.9300
O4—H4	0.8200	C16—C17	1.404 (3)
O5—C32	1.262 (4)	C16—C21	1.432 (4)
O6—C32	1.259 (4)	C17—C18	1.430 (4)
O7—C34	1.352 (4)	C18—C19	1.398 (3)
O7—H7	0.8200	C19—C22	1.401 (4)
O8—C38	1.350 (3)	C19—C20	1.439 (4)
O8—H8	0.8200	C20—C21	1.337 (5)
O9—H9A	0.8500	C20—H20	0.9300
O9—H9B	0.8500	C21—H21	0.9300
O10—H10A	0.8501	C22—C23	1.363 (4)
O10—H10B	0.8501	C22—H22	0.9300
N1—C1	1.322 (3)	C23—C24	1.396 (4)
N1—C5	1.360 (3)	C23—H23	0.9300
N2—C12	1.327 (3)	C24—H24	0.9300
N2—C6	1.362 (3)	C25—C26	1.481 (3)
N3—C13	1.331 (3)	C26—C27	1.401 (3)
N3—C17	1.351 (3)	C26—C31	1.409 (3)
N4—C24	1.322 (3)	C27—C28	1.392 (3)
N4—C18	1.362 (3)	C28—C29	1.374 (4)
C1—C2	1.391 (3)	C28—H28	0.9300
C1—H1	0.9300	C29—C30	1.375 (4)
C2—C3	1.368 (4)	C29—H29	0.9300
C2—H2	0.9300	C30—C31	1.377 (4)
C3—C4	1.392 (3)	C30—H30	0.9300
C3—H3A	0.9300	C32—C33	1.484 (4)
C4—C5	1.406 (3)	C33—C38	1.395 (4)
C4—C9	1.434 (3)	C33—C34	1.400 (4)
C5—C6	1.439 (3)	C34—C35	1.381 (6)
C6—C7	1.396 (3)	C35—C36	1.357 (6)
C7—C10	1.402 (4)	C35—H35	0.9300
C7—C8	1.430 (4)	C36—C37	1.378 (6)
C8—C9	1.345 (4)	C36—H36	0.9300
C8—H5	0.9300	C37—C38	1.384 (4)
C9—H9	0.9300	C37—H37	0.9300
			
O9—Mn1—N3	88.56 (8)	C15—C14—C13	119.9 (3)
O9—Mn1—N1	97.05 (7)	C15—C14—H14	120.1
N3—Mn1—N1	93.35 (7)	C13—C14—H14	120.1
O9—Mn1—N2	91.40 (7)	C14—C15—C16	119.9 (3)
N3—Mn1—N2	172.76 (7)	C14—C15—H15	120.0
N1—Mn1—N2	79.47 (7)	C16—C15—H15	120.0
O9—Mn1—N4	163.43 (7)	C17—C16—C15	116.9 (3)
N3—Mn1—N4	79.31 (8)	C17—C16—C21	118.1 (3)
N1—Mn1—N4	94.91 (7)	C15—C16—C21	125.0 (3)
N2—Mn1—N4	102.05 (7)	N3—C17—C16	122.5 (3)
O9—Mn1—O1	89.90 (6)	N3—C17—C18	117.1 (2)
N3—Mn1—O1	94.82 (7)	C16—C17—C18	120.4 (2)
N1—Mn1—O1	169.40 (7)	N4—C18—C19	122.8 (2)
N2—Mn1—O1	92.41 (7)	N4—C18—C17	117.2 (2)
N4—Mn1—O1	80.02 (7)	C19—C18—C17	120.0 (2)
C25—O1—Mn1	134.25 (15)	C18—C19—C22	117.0 (3)
C27—O3—H3	109.5	C18—C19—C20	118.1 (3)
C31—O4—H4	109.5	C22—C19—C20	124.9 (3)
C34—O7—H7	109.5	C21—C20—C19	121.8 (3)
C38—O8—H8	109.5	C21—C20—H20	119.1
Mn1—O9—H9A	98.5	C19—C20—H20	119.1
Mn1—O9—H9B	120.2	C20—C21—C16	121.4 (3)
H9A—O9—H9B	117.3	C20—C21—H21	119.3
H10A—O10—H10B	100.1	C16—C21—H21	119.3
C1—N1—C5	117.2 (2)	C23—C22—C19	120.2 (3)
C1—N1—Mn1	129.29 (16)	C23—C22—H22	119.9
C5—N1—Mn1	113.44 (14)	C19—C22—H22	119.9
C12—N2—C6	117.3 (2)	C22—C23—C24	119.0 (3)
C12—N2—Mn1	129.62 (17)	C22—C23—H23	120.5
C6—N2—Mn1	112.86 (14)	C24—C23—H23	120.5
C13—N3—C17	118.6 (2)	N4—C24—C23	122.8 (3)
C13—N3—Mn1	127.83 (19)	N4—C24—H24	118.6
C17—N3—Mn1	113.54 (16)	C23—C24—H24	118.6
C24—N4—C18	118.2 (2)	O2—C25—O1	122.8 (2)
C24—N4—Mn1	128.75 (17)	O2—C25—C26	119.8 (2)
C18—N4—Mn1	112.31 (15)	O1—C25—C26	117.5 (2)
N1—C1—C2	123.4 (2)	C27—C26—C31	117.6 (2)
N1—C1—H1	118.3	C27—C26—C25	122.2 (2)
C2—C1—H1	118.3	C31—C26—C25	120.2 (2)
C3—C2—C1	119.3 (2)	O3—C27—C28	117.5 (2)
C3—C2—H2	120.4	O3—C27—C26	121.5 (2)
C1—C2—H2	120.4	C28—C27—C26	121.0 (3)
C2—C3—C4	119.7 (2)	C29—C28—C27	118.8 (3)
C2—C3—H3A	120.2	C29—C28—H28	120.6
C4—C3—H3A	120.2	C27—C28—H28	120.6
C3—C4—C5	117.0 (2)	C28—C29—C30	122.2 (3)
C3—C4—C9	124.0 (2)	C28—C29—H29	118.9
C5—C4—C9	119.0 (2)	C30—C29—H29	118.9
N1—C5—C4	123.4 (2)	C29—C30—C31	118.9 (3)
N1—C5—C6	116.98 (19)	C29—C30—H30	120.6
C4—C5—C6	119.6 (2)	C31—C30—H30	120.6
N2—C6—C7	123.5 (2)	O4—C31—C30	117.8 (3)
N2—C6—C5	116.87 (19)	O4—C31—C26	120.8 (2)
C7—C6—C5	119.7 (2)	C30—C31—C26	121.5 (3)
C6—C7—C10	117.3 (2)	O6—C32—O5	123.7 (3)
C6—C7—C8	119.3 (2)	O6—C32—C33	117.8 (3)
C10—C7—C8	123.4 (2)	O5—C32—C33	118.5 (3)
C9—C8—C7	121.3 (2)	C38—C33—C34	118.2 (3)
C9—C8—H5	119.4	C38—C33—C32	120.0 (3)
C7—C8—H5	119.4	C34—C33—C32	121.7 (3)
C8—C9—C4	121.0 (2)	O7—C34—C35	119.0 (4)
C8—C9—H9	119.5	O7—C34—C33	120.3 (4)
C4—C9—H9	119.5	C35—C34—C33	120.7 (4)
C11—C10—C7	119.3 (2)	C36—C35—C34	119.3 (4)
C11—C10—H10	120.3	C36—C35—H35	120.3
C7—C10—H10	120.3	C34—C35—H35	120.3
C10—C11—C12	119.8 (2)	C35—C36—C37	122.3 (4)
C10—C11—H11	120.1	C35—C36—H36	118.9
C12—C11—H11	120.1	C37—C36—H36	118.9
N2—C12—C11	122.8 (2)	C36—C37—C38	118.4 (4)
N2—C12—H12	118.6	C36—C37—H37	120.8
C11—C12—H12	118.6	C38—C37—H37	120.8
N3—C13—C14	122.1 (3)	O8—C38—C37	118.4 (3)
N3—C13—H13	118.9	O8—C38—C33	120.5 (3)
C14—C13—H13	118.9	C37—C38—C33	121.1 (3)
			
O9—Mn1—O1—C25	−13.4 (2)	C10—C11—C12—N2	−1.2 (4)
N3—Mn1—O1—C25	−101.9 (2)	C17—N3—C13—C14	−0.1 (4)
N1—Mn1—O1—C25	117.7 (4)	Mn1—N3—C13—C14	178.8 (2)
N2—Mn1—O1—C25	78.0 (2)	N3—C13—C14—C15	2.3 (5)
N4—Mn1—O1—C25	179.8 (2)	C13—C14—C15—C16	−1.3 (5)
O9—Mn1—N1—C1	−95.6 (2)	C14—C15—C16—C17	−1.6 (4)
N3—Mn1—N1—C1	−6.7 (2)	C14—C15—C16—C21	178.3 (3)
N2—Mn1—N1—C1	174.2 (2)	C13—N3—C17—C16	−3.1 (4)
N4—Mn1—N1—C1	72.9 (2)	Mn1—N3—C17—C16	177.90 (19)
O1—Mn1—N1—C1	133.7 (4)	C13—N3—C17—C18	176.9 (2)
O9—Mn1—N1—C5	87.70 (15)	Mn1—N3—C17—C18	−2.1 (3)
N3—Mn1—N1—C5	176.66 (15)	C15—C16—C17—N3	3.9 (4)
N2—Mn1—N1—C5	−2.42 (14)	C21—C16—C17—N3	−176.1 (2)
N4—Mn1—N1—C5	−103.79 (15)	C15—C16—C17—C18	−176.1 (2)
O1—Mn1—N1—C5	−42.9 (4)	C21—C16—C17—C18	4.0 (4)
O9—Mn1—N2—C12	82.4 (2)	C24—N4—C18—C19	−0.7 (3)
N1—Mn1—N2—C12	179.3 (2)	Mn1—N4—C18—C19	−171.54 (18)
N4—Mn1—N2—C12	−87.9 (2)	C24—N4—C18—C17	178.9 (2)
O1—Mn1—N2—C12	−7.6 (2)	Mn1—N4—C18—C17	8.1 (2)
O9—Mn1—N2—C6	−91.84 (15)	N3—C17—C18—N4	−4.1 (3)
N1—Mn1—N2—C6	5.07 (14)	C16—C17—C18—N4	175.9 (2)
N4—Mn1—N2—C6	97.90 (15)	N3—C17—C18—C19	175.5 (2)
O1—Mn1—N2—C6	178.21 (15)	C16—C17—C18—C19	−4.5 (3)
O9—Mn1—N3—C13	17.3 (2)	N4—C18—C19—C22	1.6 (4)
N1—Mn1—N3—C13	−79.7 (2)	C17—C18—C19—C22	−178.1 (2)
N4—Mn1—N3—C13	−174.1 (2)	N4—C18—C19—C20	−178.3 (2)
O1—Mn1—N3—C13	107.1 (2)	C17—C18—C19—C20	2.1 (3)
O9—Mn1—N3—C17	−163.78 (16)	C18—C19—C20—C21	0.9 (4)
N1—Mn1—N3—C17	99.25 (17)	C22—C19—C20—C21	−179.0 (3)
N4—Mn1—N3—C17	4.88 (16)	C19—C20—C21—C16	−1.4 (5)
O1—Mn1—N3—C17	−74.00 (16)	C17—C16—C21—C20	−1.0 (4)
O9—Mn1—N4—C24	−132.9 (3)	C15—C16—C21—C20	179.0 (3)
N3—Mn1—N4—C24	−176.5 (2)	C18—C19—C22—C23	−1.1 (4)
N1—Mn1—N4—C24	91.0 (2)	C20—C19—C22—C23	178.8 (3)
N2—Mn1—N4—C24	10.7 (2)	C19—C22—C23—C24	−0.1 (4)
O1—Mn1—N4—C24	−79.6 (2)	C18—N4—C24—C23	−0.6 (4)
O9—Mn1—N4—C18	36.7 (3)	Mn1—N4—C24—C23	168.5 (2)
N3—Mn1—N4—C18	−6.94 (15)	C22—C23—C24—N4	1.0 (4)
N1—Mn1—N4—C18	−99.43 (15)	Mn1—O1—C25—O2	11.5 (4)
N2—Mn1—N4—C18	−179.69 (15)	Mn1—O1—C25—C26	−168.17 (15)
O1—Mn1—N4—C18	89.96 (15)	O2—C25—C26—C27	178.5 (2)
C5—N1—C1—C2	1.5 (4)	O1—C25—C26—C27	−1.8 (3)
Mn1—N1—C1—C2	−175.08 (19)	O2—C25—C26—C31	−0.7 (3)
N1—C1—C2—C3	−3.0 (4)	O1—C25—C26—C31	179.0 (2)
C1—C2—C3—C4	1.1 (4)	C31—C26—C27—O3	−178.9 (2)
C2—C3—C4—C5	2.0 (4)	C25—C26—C27—O3	1.9 (3)
C2—C3—C4—C9	−178.5 (2)	C31—C26—C27—C28	1.6 (3)
C1—N1—C5—C4	2.0 (3)	C25—C26—C27—C28	−177.6 (2)
Mn1—N1—C5—C4	179.11 (17)	O3—C27—C28—C29	178.5 (3)
C1—N1—C5—C6	−177.6 (2)	C26—C27—C28—C29	−1.9 (4)
Mn1—N1—C5—C6	−0.5 (2)	C27—C28—C29—C30	0.7 (5)
C3—C4—C5—N1	−3.8 (3)	C28—C29—C30—C31	0.8 (5)
C9—C4—C5—N1	176.8 (2)	C29—C30—C31—O4	177.9 (3)
C3—C4—C5—C6	175.9 (2)	C29—C30—C31—C26	−1.2 (4)
C9—C4—C5—C6	−3.6 (3)	C27—C26—C31—O4	−179.0 (2)
C12—N2—C6—C7	−0.8 (3)	C25—C26—C31—O4	0.2 (4)
Mn1—N2—C6—C7	174.24 (17)	C27—C26—C31—C30	0.0 (4)
C12—N2—C6—C5	178.0 (2)	C25—C26—C31—C30	179.2 (2)
Mn1—N2—C6—C5	−7.0 (2)	O6—C32—C33—C38	177.9 (3)
N1—C5—C6—N2	5.1 (3)	O5—C32—C33—C38	−0.2 (4)
C4—C5—C6—N2	−174.52 (19)	O6—C32—C33—C34	1.2 (4)
N1—C5—C6—C7	−176.0 (2)	O5—C32—C33—C34	−176.9 (3)
C4—C5—C6—C7	4.3 (3)	C38—C33—C34—O7	−177.9 (3)
N2—C6—C7—C10	−1.8 (3)	C32—C33—C34—O7	−1.2 (4)
C5—C6—C7—C10	179.5 (2)	C38—C33—C34—C35	0.2 (4)
N2—C6—C7—C8	177.0 (2)	C32—C33—C34—C35	176.9 (3)
C5—C6—C7—C8	−1.7 (3)	O7—C34—C35—C36	179.2 (4)
C6—C7—C8—C9	−1.6 (4)	C33—C34—C35—C36	1.0 (6)
C10—C7—C8—C9	177.1 (2)	C34—C35—C36—C37	−1.3 (6)
C7—C8—C9—C4	2.3 (4)	C35—C36—C37—C38	0.2 (6)
C3—C4—C9—C8	−179.1 (2)	C36—C37—C38—O8	−178.2 (3)
C5—C4—C9—C8	0.3 (3)	C36—C37—C38—C33	1.1 (5)
C6—C7—C10—C11	2.8 (4)	C34—C33—C38—O8	178.0 (3)
C8—C7—C10—C11	−175.9 (2)	C32—C33—C38—O8	1.2 (4)
C7—C10—C11—C12	−1.5 (4)	C34—C33—C38—C37	−1.3 (4)
C6—N2—C12—C11	2.3 (3)	C32—C33—C38—C37	−178.1 (3)
Mn1—N2—C12—C11	−171.75 (19)		

### Hydrogen-bond geometry (Å, º)

**Table:**

*D*—H···*A*	*D*—H	H···*A*	*D*···*A*	*D*—H···*A*
O3—H3···O1	0.82	1.83	2.552 (2)	147
O4—H4···O2	0.82	1.79	2.525 (3)	148
O7—H7···O6	0.82	1.79	2.518 (4)	147
O8—H8···O5	0.82	1.74	2.474 (3)	149
O9—H9*A*···O2	0.85	1.90	2.735 (2)	169
O9—H9*B*···O6	0.85	1.80	2.648 (3)	171
O10—H10*A*···O5	0.85	2.07	2.890 (3)	162
O10—H10*B*···O5^i^	0.85	2.09	2.890 (3)	156

Symmetry code: (i) −*x*+2, *y*, −*z*+1/2.
